# Multiplying Meanings of Pregnancy Through Personal Accounts of Gestational Trophoblastic Disease

**DOI:** 10.1177/1357034X241286356

**Published:** 2024-10-28

**Authors:** Emily Ross

**Affiliations:** University of Sheffield

**Keywords:** embodiment, gestational trophoblastic disease, hormones, human chorionic gonadotrophin (hCG), pregnancy

## Abstract

In cultures where reproduction is highly medicalised, pregnancy is often understood in terms of foetal development and an anticipated baby. This is connected to a wider privileging of the ‘foetal subject’ in these settings, which has had implications for reproductive autonomy. In this article, I disrupt dominant understandings of pregnancy by engaging with qualitative accounts of gestational trophoblastic disease. This rare condition can entail experiences of pregnancy without foetal development, allowing for scholarly attention to the wider biological, affective and relational constituents of this corporeal event. In this article, I pay particular attention to the ‘pregnancy hormone’ human chorionic gonadotropin (hCG), which in the context of gestational trophoblastic disease becomes a biomarker for disease. My research extends feminist science studies perspectives destabilising understandings of maternal and foetal bodies as bounded and distinct entities. The article de-centres foetal development as the most significant consequence of conception and enriches feminist discussions of reproductive politics.

## Introduction

[My doctor said] even though the pregnancy is no longer technically going on, placental growth is continuing. And her explaining, you know, you’re still producing those hormones . . . at least was a little bit of a comfort to know . . . well, why do I still feel pregnant if I’m not? (Bea)

This week my hCG hormones have increased. Even after two surgeries, my body still thinks I am pregnant. (Joan)

What makes a body pregnant? These narratives are voiced by women affected by gestational trophoblastic disease, a rare placental condition which prohibits viable foetal development. In their accounts, ‘feeling’ pregnant is attributed to placental growth and the production of hormones. Their corporeal experiences unsettle dominant representations of pregnancy. Within antenatal care, popular culture and personal lives, understandings of pregnancy equate it with an anticipated baby. Reflecting this, though Bea described ‘feeling’ pregnant her clinician positions her as not ‘technically’ so, because she has had surgery to remove the products of conception.

Descriptions of pregnancy in these varying degrees – as being ‘technically pregnant’ or as the body ‘thinking’ it is pregnant – point to a multiplicity that is not captured by the singular version of pregnancy prevalent in biomedicalised societies. This version emphasises foetal development and anticipated foetal futures, with the birth of a baby framed as pregnancy’s natural endpoint (Browne, 2022a). As well as silencing lived experiences of pregnancy which do not end in this way (Layne, 2003), this view shapes clinical and regulatory practices of reproduction, with implications for reproductive autonomy (Beynon-Jones, 2012). In this article, I draw on patient and practitioner accounts of gestational trophoblastic disease to disrupt dominant representations of pregnancy. Inspired by scholars attending to hormones as a way to unsettle established categories of reproductive experience, I do this through particular attention to the ‘pregnancy hormone’ human chorionic gonadotropin (hCG). My exploration of gestational trophoblastic disease allows me to foreground the corporeal, material and relational elements that constitute contemporary experiences of pregnancy, but which remain overshadowed by bioscientific accounts of foetal development in clinical management and in law.

### Feminist Engagement With the Biological (Pregnant) Body

Feminist authors have mobilised embodied accounts of pregnancy to interrogate Western philosophical individualism and perceptions of the unified subject. These perspectives have been challenged by feminist authors because they are predicated upon masculinist ideals of autonomy and singularity, disregarding the fluidity, relationality and ‘leakiness’ of (feminine) experience (Kilby and Lury, 2000). They have also contributed to socio-legal framings of pregnancy as entailing two distinct entities, with these at times positioned as oppositional (Shildrick, 2022). In an early critique, Young’s (1984) personal account pointed to gestation as a decentring or doubling, transcending dualisms between the body as subject and as object as she experiences foetal movements as ‘wholly mine . . . conditioning my experience and space’ (p. 48). Such accounts of pregnant embodiment have been crucial in challenging depictions of reproduction as involving separate and conflicting individuals (Tyler, 2000). The representation of gestating and foetal bodies as independent beings has been exacerbated by the biomedicalization of reproduction and related emergence of the ‘foetal subject’, facilitated by obstetric imaging technologies and clinical procedures such as foetal surgery (Casper, 1998; Shaw, 2012). The sociocultural solidification of the foetus as a, if not *the*, subject of pregnancy has profoundly shaped reproductive care in biomedicalised societies (Duden, 1993), with potential foetal futures considered equally or privileged over gestating bodies across regulatory and healthcare settings (Beynon-Jones, 2012; Markens et al., 1997).

In recent years, feminist perspectives have mobilised bioscientific accounts of the material body to trouble deterministic and dualistic representations of gestation, showing that meaningful engagement with the biological can encourage more generous and caring responses to this event (Hird, 2007). This has coincided with a wider interest within feminist thought in the new materialisms, described by Yoshizawa (2016) as work that ‘identifies and mobilizes developments within the natural sciences’ in support of feminist attempts to ‘theorize naturecultures, inter- and intra-corporeality, and reproductive politics’ (p. 83). For example, work has explored the phenomenon of microchimerism, the bidirectional ‘trafficking’ of cells between maternal and foetal bodies, as a way to dismantle the imagined boundaries between the bodies of gestation (Kelly, 2012; Martin, 2010), with wider implications for how the individualised subject is understood within biomedical, legal, philosophical and political discourse (Shildrick, 2022). Yoshizawa (2016) reconsiders dominant narratives of the placenta, which generally position this organ as uniting discrete maternal and foetal bodies. Citing new materialist work and emerging scientific research on microchimerism and immunology, Yoshizawa instead frames this relationship as one of *intra*-action, whereby ‘mother, foetus and placenta’ are seen as mutually becoming. These perspectives reconfigure the relationships between gestating bodies, their environments and over time, and widen responsibilities for reproductive outcomes beyond those who are pregnant (Lappé and Hein, 2023; Yoshizawa, 2016).

With similar theoretical foundations, feminist scholars have considered bioscientific representations of hormones. Commonly depicted as ‘messengers’ of predetermined signals such as those relating to sexual difference (Oudshoorn, 1995) or stress (Roberts and McWade, 2021), authors have complicated understandings of these chemical actors as expressions of predetermined biological phenomena. They show instead that hormones function within biosocial worlds which shape their representation and hormonal experience (Roberts, 2007). Engaging with histories and sociocultural narratives, this work demonstrates how particular framings of hormones constitute contemporary understandings of human experiences such as sex or reproduction, with hormones always actively participating ‘in the enactment of particular versions’ of the biological and the social (Roberts, 2007: xv). By understanding hormones in alternative ways, as messaging *across* these domains (Roberts and McWade, 2021), we can break down the distinctions that commonly order understandings of areas of life including reproduction. This approach to scientific knowledge destabilises the biological ‘facts’ of reproducing bodies, making way for more liberating versions of these experiences.

In this article, I am inspired by authors engaging with bioscientific objects to challenge the notion of (gestating) bodies ‘as discrete entities, clearly bounded and differentiated’ (Blackman, 2010: 1), particularly where this has implications for the politics of reproduction. However, while several scholars have engaged with corporeal elements of pregnancy to reframe relationships between the bodies of gestation, this is often through discussion of pregnancy in terms of foetal development. I extend existing perspectives by examining a pregnancy-related event that has not yet been substantively considered in feminist literature: gestational trophoblastic disease. This rare condition follows conception but does not entail the development of a viable foetus or, in some cases, any foetal development at all.

### Gestational Trophoblastic Disease: A Social Scientific Perspective

Feminist perspectives which mobilise bioscientific accounts have been hugely influential in reframing the relationships between maternal/foetal bodies. However, by predominantly engaging with the physiology of continuing pregnancies that (are anticipated to) entail ‘successful’ foetal development, this work does not challenge dominant representations of pregnancies ‘that end in “normal” live births as the norm’ (DiCaglio, 2017: 3). DiCaglio (2018) calls for increased attention to *all* stages and forms of reproduction, including those which do not result in term-birth. Referencing recent work on placental biology, she encourages feminist theory to account not just for the placenta as an established organ but for the *developing* placenta. Engaging with the biology of pregnancy at a stage prior to the development of what may or may not become foetal tissues reconfigures the bodies, boundaries and relationships of gestation. By centring development rather than birth, feminist perspectives can therefore make room for pregnancy’s multiplicity – including when this ends in loss. Turning attention away from the anticipated future product of pregnancy, and instead towards those aspects of pregnancy that ‘ring true no matter what’, such as microchimeric relations, DiCaglio argues that we might consider pregnancy as ‘a project unfolding in time, representing many potential processes, outcomes and developmental turns’ (p. 284).

DiCaglio’s contribution joins existing work destabilising the ‘teleological’ model of foetal development (Franklin, 1991). This conceptualisation is focussed on the anticipated endpoint of pregnancy, and today dominates regulatory and wider social discourse surrounding reproduction in the present (Beynon-Jones, 2017; Franklin 2014). In her recent monograph, Browne (2022a) argues that attending to pregnancies which do not end as anticipated, what she calls ‘pregnancy without birth’, also has implications for lived experiences of loss. Browne calls for feminist scholars to push beyond dominant discourses of pregnancy loss which centre ‘normal’ and ‘natural’ pregnancies and which reinforce future-oriented understandings of this corporeal event. This can challenge the idea that ‘productive’ pregnancy is the only pregnancy that counts (p. 19), with implications for the articulations of failure, guilt and self-blame often (though not always) associated with experiences of miscarriage.

In this article, I add to this growing body of work by presenting accounts of gestational trophoblastic disease. This condition arises from trophoblastic cells, which in the majority of pregnancies develop into a large part of the placenta (Wang and Zhao, 2010). Due to chromosomal faults at conception, trophoblastic cells proliferate excessively in gestational trophoblastic disease, and any developing foetal tissue will either be non-viable or entirely absent. The most common form is known as molar pregnancy, with the majority of these successfully treated with surgery and sometimes chemotherapy. Rarely, trophoblastic tissue can become cancerous (choriocarcinoma) and require more intensive treatment (Seckl et al., 2010).

Gestational trophoblastic disease is of significance to social scientists of reproduction because it provides an opportunity to engage with embodied elements of pregnancy in the absence of viable foetal development, the aspect of pregnancy so often centred in biomedicine, clinical care and personal experience. Molar pregnancies in particular offer an opportunity to engage with what Browne calls ‘pregnancy without birth’, but as I show, extend this work by interrogating the very meaning of this event: what could be conceptualised as a ‘pregnancy without pregnancy’. For example, like any other pregnancy, molar pregnancies begin with a conception which prompts implantation, amenorrhea, increased progesterone and, significantly, the production of the hormone hCG (Cole, 2010). In this article, I demonstrate that these processes could be articulated as rendering bodies as pregnant without (viable) foetal development, exposing multiplicities and contingencies of this apparently unequivocal bodily event.

The experiences I draw on below have been gathered for a sociological research project on gestational trophoblastic disease. The aim of this work was to use this rare disease as a lens to interrogate dominant sociocultural framings of both pregnancy and cancer, exploring their role in the mutual shaping of the disease, patient experience and clinical work. Interviews and observations took place across sites including the clinic, the laboratory and within online spaces.^1^ Due to a concern with corporeal accounts, patient experiences (all pseudonymised) formed the bulk of data. These were accessed through long narrative accounts of partial molar pregnancy, complete molar pregnancy and choriocarcinoma recorded in 22 online blogs and in 13 semi-structured interviews (eight were conducted with blog authors). Extracts from blog posts are anonymised and presented verbatim with consent, but where consent could not be obtained these are paraphrased. Importantly, these women all welcomed the pregnancies later diagnosed as gestational trophoblastic disease. Interviews with 11 healthcare professionals, observations of multidisciplinary team meetings and laboratory observations at two of the three UK specialist centres were also conducted. The multi-sited nature of this project enabled a holistic appreciation of the elements that enable gestational trophoblastic disease to be understood, experienced and managed as it is. It also provided insight into the technologies, bodily substances and care practices that contribute to this, and how its biomedical location between reproduction and disease impacts patient experience.

In what follows I present patient narratives of diagnosis and monitoring in the contexts of pregnancy/gestational trophoblastic disease (with these experiences entwined), focussing particularly on articulations of the hormone hCG. I introduce patients’ accounts of hCG testing in early pregnancy, and their interpretations of the meaning of hCG in this context, before moving on to how these meanings could both shift and stick as the hormone became experienced as a biomarker for disease. Finally, I show how attention to embodied experiences of gestational trophoblastic disease, as well as its management in clinical practice, allows us to understand pregnancy as taking place beyond foetal development. My findings destabilise singular, teleological models of pregnancy, and joining DiCaglio (2018) and Browne (2022a), de-privilege livebirth as the only significant consequence of conception.

## Becoming Pregnant: Materialising hCG

hCG is colloquially termed ‘the pregnancy hormone’, so named because it is the chemical detected by home and clinically administered pregnancy tests. Scientific literature describes the hormone as driving implantation, supporting the developing embryo and as critical to establishing and sustaining a successful pregnancy (Cole, 2012b). This is through processes including ‘immune regulation at the maternal/foetal interface’ and enabling the ‘maternal recognition’ of pregnancy (d’Hauterive et al., 2022; Gridelet et al., 2020). Here, scientific narratives of hCG resonate with those of the placenta, which position placenta, mother and foetus as separate yet ‘interfacing’ entities (Yoshizawa, 2016). hCG performs important explanatory work in these scientific accounts, with its function and purpose depicted as driven by the embryo and geared towards foetal development. This accords with and also shapes sociocultural understandings of pregnancy which centre the foetal subject as the primary and inevitable outcome of gestation. However, engaging with bioscientific accounts more closely provides the opportunity to disrupt this framing. For example, this representation obscures the fact that a positive result for hCG does not signify the presence of a developing foetus but of placental tissue (Olszynko-Gryn, 2014). Furthermore, at least five variants of the molecule have been identified, each with different physiological structures and functions, including in some cancers (Cole, 2012a). These nuances are collapsed in representations of hCG as ‘the’ pregnancy hormone. Understandings of hCG as indicative of a pregnancy, and by extension a future baby, have important consequences for how we interpret its meaning. As noted by Layne (2009) and Han (2014), testing for the presence or absence of this hormone, which organises some bodies as pregnant and others as not, reduces the complex physiological changes that follow conception to a single chemical. hCG testing does not account for the early weeks following conception, too early for detection by many home tests. It also represents non-viable pregnancies, including chemical, molar and anembryonic, as analogous to those which have the potential for a livebirth, visually depicting these diverse events with the same two lines, or through the word ‘Pregnant’ in the case of digital tests.

Perceptions of a positive hCG result as marking the start of a pregnancy were articulated by participants in this research. Most equated a positive test result with a hoped-for future baby. Helen wrote in her blog that from the moment she saw two pink lines she felt like she ‘had a baby’, and was just ‘waiting for him to be born’. Cath similarly wrote about ‘falling in love’ on seeing the ‘lines on the test’. The result itself could be sufficient for some to make material changes including decorating a nursery, or mental preparations for a ‘big life change’, as Iris and Annie reported in their interviews. Due to the high levels of hCG produced in molar pregnancy, the test can return a strong positive, which provided some with more certainty in designating a pregnancy. Barbara wrote that she had never seen ‘such solid red lines’ before. She compared this to the faint result received with her first pregnancy, which therefore had not been ‘confirmed’ until she saw her gynaecologist.

Though participants’ stories demonstrated the significance of hCG to rendering them pregnant, some disrupted the seemingly straightforward equation of hCG’s presence with a developing baby. For some this was more tentative, contingent upon further testing or the passing of time as they waited to confirm foetal development visually through ultrasound (see also Ross, 2018). Bea interpreted her positive result in light of physical symptoms, comparing these to a prior pregnancy. For her, a previous loss inspired a more complex emotional engagement with the positive result. Nevertheless, the result was sufficient to prompt feelings of love and care towards a hoped-for baby:

I found out I was pregnant with you early on a Saturday morning. It was technically a day too early to test, but the night before I had taken a bite of cake and it tasted off. This paired with a slightly queasy feeling made me take the test, smiling when the appearance of a second line proved my suspicions right . . . With my hand over my stomach, I thought about how next Christmas would be your first . . . Even though I was queasy, even though I was sometimes afraid, we loved you. (Bea, blog)

Many women’s stories of pregnancy testing situated their reading of a positive hCG result within long-held hopes for a child. In her interview, Diana explained this had contributed to her envisaging a future baby’s ‘whole lifetime’ on discovering she was pregnant. Interpretations could also be configured by more immediate personal situations:

When you see those two lines on the pregnancy test, your hope is there right? Like you can’t help but like your mind starts racing nine months from now . . . I was like it’s great, the baby’s going to come in [Spring], like the children’s school will be done, it’s the perfect time. (Joan, interview)

Here, Joan’s imagination and anticipation for a future baby were shaped by how it would fit within her existing family. The equation of hCG with a future baby was therefore multifaceted, interpreted in light of familiar bodily signs of pregnancy, such as changes to taste and nausea, and couched within participants’ family contexts, as well as wider sociocultural narratives of pregnancy. Significantly, these participants welcomed news of a pregnancy. Engagements with hCG as materialised on the pregnancy test can differ in alternative contexts such as where this leads to an abortion, a situation where dominant narratives of foetal subjecthood and obstetric technologies can be subverted (Beynon-Jones, 2015). The technology of pregnancy testing does not account for this distinction, universalising this experience by suggesting that pregnancy is a ‘single thing’ (Layne, 2009: 66) and stifling the varied responses to and outcomes that follow conception but do not lead to a live birth.

Significantly, these accounts are all from women for whom viable foetal growth was not a possibility. Nevertheless, for many, the presence of hCG as materialised on a home pregnancy test prompted anticipations for foetal development and beyond. Their experiences were not just affective but could have material impacts on the world (see also Layne, 2003). Such understandings of hCG, I argue, are shaped by teleological models of pregnancy which centre its anticipated outcome, but also the cultural significance and familiarity of home pregnancy testing. However, participant accounts have also shown the extent to which meanings of the hCG result were situated within personal circumstances, embodied experience and emotions. In the following section, I demonstrate how meanings of hCG shaped embodied experiences of surveillance and monitoring for gestational trophoblastic disease.

## Diagnosing and Monitoring Gestational Trophoblastic Disease

Following a positive pregnancy test, patient experiences and clinical pathways of molar pregnancy initially mirrored those of pregnancy ending in birth. It was then often during an ultrasound scan, either routine or prompted by symptoms such as bleeding, that patients received the news that the pregnancy would not continue and required a surgical evacuation (a D&C). Many recounted this as a time of uncertainty as sonographers who were not familiar with gestational trophoblastic disease sought second opinions or offered a tentative diagnosis. For example, in her blog, Barbara recalled her gynaecologist telling her ‘I don’t think you are pregnant’. Fiona was told ‘Your baby has no heartbeat, and it wasn’t a miscarriage’. The inability to determine a body as pregnant or miscarrying through ultrasound demonstrates the contingency of this event even when assessed by clinical tools. On learning that there had been no foetal development, or that this had ceased weeks prior, participants struggled to make sense of the corporeal and emotional experiences that had followed their positive hCG result. In her blog, Joan described that despite a scan showing no heartbeat, her body continued to ‘grow, produce hormones and give me enough nausea to throw up every night’, indicating a feeling of dissonance between the clinically determined end of her pregnancy and her ongoing bodily experience. This continued following surgery, after which she wrote ‘Although my belly is still swollen, I feel newly hollow and empty’.

Uncertainties surrounding their pregnant status could be prolonged as they waited for a molar pregnancy to be confirmed. This required examination of the tissues removed at surgery by a pathologist and hCG blood tests. As the hormone produced by trophoblastic cells, hCG is key to diagnosing and monitoring the condition. Levels are regularly tested and determine whether further treatment is required. hCG is therefore a key element of patient experience, with one scientist noting ‘you cannot talk about [gestational trophoblastic disease] without talking about hCG’. Unlike pregnancies that progress according to teleological models of development, participants’ encounters with hCG therefore extended well beyond the home pregnancy test. In most pregnancies, the hormone will peak at around 200,000 mIU/mL, but in gestational trophoblastic disease, levels can reach into the millions. Whereas home pregnancy tests merely detect hCG’s presence, in cases of gestational trophoblastic disease, a laboratory assay is used to deliver a quantitative result to track its rise and fall. With commercial assays only able to assess hCG within the ‘pregnancy range’ (Nodler et al., 2011: 7), in the UK specialist laboratory staff use a radioimmunoassay that has been developed in-house, sensitive enough to detect the hormone at all quantities. The radioimmunoassay is performed over two days and involves each sample undergoing a complex process of measurement and dilution to ensure a valid result, in stark contrast to the home pregnancy tests experienced by participants which had delivered a result within minutes.

hCG testing continues until a patient’s levels return to ‘normal’, with this defined as the level of a ‘non-pregnant’ person. In the UK NHS, most patients will be monitored for 6 months but in rare cases are monitored for life (Seckl et al., 2010). The presence of hCG is made tangible to patients through its collection in blood and urine, along with its clinical documentation and personal records of their levels in treatment diaries. Due in part to its accessibility through urine sampling, hCG monitoring can take place as much as weekly. For UK outpatients, the sending and receiving of urine sample pots is co-ordinated by post to their home address. The frequency of this monitoring could mean that the hormone became a constant presence. Though Juliet and Diana described this in their interviews as a ‘safety net’, some participants felt trapped in what Annie called a ‘hamster wheel’ of monitoring:

I spend half the week consumed by results and samples. It just gets to Tuesday and I start to feel okay again, happy occasionally too, and then Wednesday rolls round, results day, and all the worry begins. What are my hormones doing? Are my levels dropping? Wednesday comes and the results come in . . . It’s the constant churn of samples and results that doesn’t give you a break to start healing. (Annie, blog)

This ‘constant churn’ was shaped by the fact that it took days to run the hCG test in the laboratory, meaning that by the time she received her result another sample would soon be due. Annie did not feel able to ‘heal’ until she could re-start her efforts to conceive a much-wanted baby, though this was initially advised against by clinical teams. As one clinical professional explained, patients are asked to avoid another pregnancy during monitoring ‘because we are tracking the hCG, and if their hCG rises, we wouldn’t know whether it’s a complication from molar tissue or whether it’s a new pregnancy’. Here, the dual meanings of this hormone as a signifier of both pregnancy and disease tangibly impacted patients. The physical effects of monitoring could also have lasting impacts. In addition to urine tests, patients receiving treatment and some outpatients could undergo regular serum (blood) hCG testing. Gina had an hCG level of 247,000 mIU/mL before her surgery for a molar pregnancy and subsequently underwent ‘weekly blood draws’. She described these in her blog as ‘Hell on me. Physically and emotionally’. In her interview, Iris described feeling like a ‘pin cushion’ due to the frequency of hCG testing, which could make this monitoring unwelcome.

As social scientists have demonstrated, disease monitoring can have powerful impacts on identity and embodiment (Bell, 2013; Gillespie, 2012). In the context of gestational trophoblastic disease, the material aspects of hCG monitoring, whether this be the regular use of urine sample pots or frequent blood collection, were a key element of participants’ experiences. The intensity of monitoring extended their role as patients in embodied ways, but also as the tangible elements of these clinical practices began to encroach into the home. Significantly, because of understandings of hCG as the ‘pregnancy hormone’, its materialisation affected participants’ corporeal experience too, as they tried to reconcile the continued presence of hCG with the fact that there was ‘no baby’:

I know obviously I wasn’t pregnant, I had a D&C but that hormone was still there . . . So it was like this weird limbo where . . . you’re not pregnant but your body thinks it is and you’re trying to get to where your body thinks it’s not pregnant. (Joan, interview)

Joan described her treatment and monitoring as ‘a long first trimester’ and reflected that because her hormones were ‘still really high’ she remained ‘like a pregnant person’. This resonated with Annie’s experience, who recalled that she did not ‘get her periods back’ during this time because her hCG was so high. Annie was provided with leaflets about miscarriage following her initial diagnosis. However, because of ongoing bodily experiences of pregnancy, she felt these were not appropriate to her situation:

because my body hadn’t rejected the pregnancy, my hCG level was still elevated, was still climbing, and I’d had no bleeding, no pain . . . I was, like, well, it kind of isn’t a miscarriage then. I haven’t miscarried this pregnancy. (Annie, interview)

This differed slightly for Hope, who wrote ‘I almost feel like I have gone through another miscarriage’. However, in contrast to a previous loss, Hope positioned this miscarriage as ‘never ending’, due to the requirement for continued monitoring until her ‘numbers hit zero’. These accounts complicate societal understandings of miscarriage, widely understood as the loss of a developing foetus from the body. As others have noted, miscarriage too can be considered multiple, spanning days or weeks and entailing losses that can be ‘missed’ or ‘threatened’ (Browne, 2022b; Melo and Granne, 2020). Experiences of gestational trophoblastic disease further complicate teleological narratives of pregnancy and loss by introducing unexpected endpoints, but also by extending or suspending anticipated endpoints through the continued presence of hCG.

Dominant representations of hCG clearly impacted how these participants experienced and made sense of a diagnosis of gestational trophoblastic disease and its bodily effects. Continued monitoring could then shift what hCG signified and therefore its meaning: Lily described it changing from a hormone to be ‘so happy about’ to a ‘hormone that’s giving you cancer’. For those affected, hCG was no longer straightforwardly equated with a pregnancy. Some interpreted its presence as prompting the body to ‘think’ it was pregnant, or as Kim described it, stimulating a ‘phantom pregnancy’. This was attested to in Fliss’ blog, who described the disease as ‘cruel’ because ‘your body thinks you’re pregnant’ in the absence of a ‘healthy baby’. Though foetal development had stopped, Nat continued to experience symptoms for weeks. She described this in terms of deception:

My body tricked me for one month. I had sore boobs. That’s what I find most difficult. My body let me down. (Nat, interview)

Nat’s description of her body ‘tricking’ her is only allowed for by a vision of pregnancy which equates it with its anticipated outcome. Without continued foetal development Nat articulated that her body had let her down, rather than being able to appreciate the diverse corporeal changes she experienced as pregnancy in their own right. This was evident in others’ accounts. Anita experienced symptoms of pregnancy following its early ‘failure’, and in her blog reasoned that her ‘body had yet to catch up’. Similarly, Bea wrote that because she continued to feel nauseated and exhausted, her body ‘hadn’t gotten the memo’. Here, pregnancy is represented as a singular experience: one is either pregnant (with a baby) or they are not pregnant – though the body can *think* it is. In the absence of ongoing foetal development, these women did not represent the very real physiological and emotional changes prompted by conception as a pregnancy. Instead they qualified this as ‘like’ a pregnancy or the body ‘thinking’ it was pregnant. Their narratives privileged pregnancy in terms of its anticipated outcome, rather than as an embodied process, in ways that rendered their bodily experiences as in some way false or deceptive. This also meant that very real shifts in corporeality, as well as their emotional responses to these changes and their positive pregnancy test, could be denied by others. One clinical professional lamented that some of her patients had been told by family or friends that they ‘weren’t really pregnant’ because there ‘wasn’t a baby there’.

These personal accounts have demonstrated how dominant narratives of hCG as the ‘pregnancy hormone’ impact experiences of diagnosing and monitoring gestational trophoblastic disease. A singular view of pregnancy as equated with foetal development also shapes the bioscientific assessment of hCG, with most commercially available technologies unable to measure hCG at levels beyond those expected in a ‘typical’ pregnancy. The material practices involved in collecting hCG for analysis using the specialised laboratory assay, requiring days to process, could situate patients within a constant cycle of testing. This meant they could not easily ‘move forward’ from the pregnancy, particularly in the case of those who wanted to continue their reproductive journeys. Practices of measuring and monitoring hCG are thus a clear example of scientific tools both shaping and reproducing the social world, as Law (2017) and other Science and Technology Studies scholars have long shown. The ongoing presence of hCG also impacts embodied experiences, particularly for molar pregnancy patients who attempted to reconcile this, and in some cases associated symptoms of pregnancy, with the impossibility of a baby. Participants’ experiences of hCG monitoring encourage us to broaden our understandings of what pregnancy is and what it entails, to fully appreciate the elements of this experience beyond foetal development. These can be brought into focus through alternative patient reflections on the disease, which I discuss below.

## Beyond the Foetal Subject: Centring Pregnancy as Process

Alongside the ambiguous accounts of pregnant corporeality discussed above, alternative testimonies from patients and clinicians centred emotion and the physiological ‘process’ as defining pregnancy. The embodied elements of this were significant, particularly in the case of molar pregnancies where those affected can experience well-recognised bodily signs. Molar pregnancy patients can even undergo exaggerated symptoms of pregnancy, as was the case for Juliet who described in her interview that she had begun to experience ‘late pregnancy symptoms very early on’, including ‘boobs leaking’ and a ‘little belly’. For some, corporeal experiences led them to conceptually separate pregnancy from foetal development:

Although there never was a baby in my womb during my first pregnancy, it was still a pregnancy and it was still a loss. (Diana, blog)

For me there wasn’t a baby, but I was still pregnant. (Annie, interview)

Annie, who received a provisional diagnosis of complete molar pregnancy at her 12-week ultrasound scan, described gestational trophoblastic disease as the ‘loss of her pregnancy’. This loss did not entail a knowable entity that could be grieved: indeed, she described that because there was ‘no baby’ she could more easily ‘detach’ herself emotionally. What was lost was ‘three months of being pregnant’, which for her had begun with a positive hCG result and entailed consequences for her corporeality, including changes to her hair and skin, amenorrhea, nausea, bloating and ‘mood swings’. These women discussed pregnancy beyond an anticipated baby, as a whole-body event with lasting legacies. Others described their experience of pregnancy in terms of the plans they had made or suspended to accommodate its anticipated endpoint. As we saw above, for many, these plans began on attaining a positive test result. Elaine blogged about her experience as a feeling of grief for these ‘dreams, plans and hopes’, despite the absence of a ‘developing child’. She explained that she had been ‘pregnant like any other woman’ and had ‘lost her baby’. This was also expressed by Juliet:

Although it wasn’t an actual human life there, I think, it was a dream, do you know what I mean? It was, like, we were so excited to have another child . . . in your mind you, kind of, plan out what’s going to happen here, and what’s going to happen there. So, I think the loss of that dream, rather than the loss of the, it, it is quite affecting. (Juliet, interview)

This quote describes the constitution of Juliet’s pregnancy through ‘dreams, plans and hopes’ the reality of which for participants, but also their loved ones, did not necessitate the physical presence of a ‘developing child’.

The significance of imagination and emotion to constituting lived pregnancy was well-known by the clinical professionals I interviewed and meant that they often foregrounded patients’ accounts in the definitions of pregnancy that guided their work. A gynaecologist explained that patients ‘see a molar pregnancy as a pregnancy just like a non-molar pregnancy, and they look at it as a pregnancy loss’. Within his clinical practice he was ‘mindful that we treat it as though they are’. Another clinical professional described that when a patient seeks clarification of whether she had experienced a pregnancy, she answers that they were: ‘yeah, you know, ‘cause that’s all your hopes and dreams like, just been snatched away in a second’. The definitions of pregnancy advanced by these practitioners, and others I interviewed, emphasise embodiment and emotion, with this determining clinical pathways. Even in cases of a complete molar pregnancy, which the pathologist I interviewed did not consider a pregnancy but a ‘tumour’, patients were offered ongoing support and counselling that mirrored other forms of reproductive loss.

The formulation of dreams and plans featuring an imagined baby or child is reflective of the orientation towards the future that characterises biomedicalised societies (Adams et al., 2009) and is particularly evident in the management of reproduction (Ballif, 2023). Indeed, initial engagement with clinical care, which entailed the measurement and forecasting of participants’ pregnancies according to ‘gestational age’, could be another key setting for the designation of their status as pregnant, as well as the formation of expected outcomes. In biomedicalised societies, pregnancy is overwhelmingly equated with foetal development and the anticipated birth of a baby. The personal accounts of gestational trophoblastic disease presented above have shown instead that pregnancy can be located throughout the body, in the corporeal shifts prompted by conception and through the ongoing presence of hCG. Pregnancy was also constituted beyond the body (see Beynon-Jones, 2017), for example, through the affective work provoked by a positive pregnancy test. These insights contribute to feminist endeavours to de-centre the foetal subject and destabilise representations of pregnancy according to teleological perspectives. I discuss this further below.

## Multiplying Meanings of Pregnancy

The Introduction to this article asked ‘what makes a body pregnant?’. In biomedicalised societies gestation is managed and understood according to a teleological view, with its anticipated outcome shaping prenatal clinical management, the regulation of scientific practice and access to reproductive healthcare (Franklin, 1991). This has also been shaped by and contributes to foetal-centric framings of reproduction beyond biomedicine, enabled in part by medical and technological intervention including obstetric ultrasound, but also by the everyday routines and rituals undertaken by those who are pregnant and their families (Han, 2013). Feminist science studies scholars have engaged with the physiology of pregnancy to successfully trouble dualistic understandings of gestating and foetal bodies, showing these to be mutually constitutive (Martin, 2010; Yoshizawa, 2016). This allows for a more generous and caring representation of pregnancy, where these bodies are understood to be in a symbiotic rather than adversarial relationship (Hird, 2007). However, as DiCaglio (2018) has noted, such work maintains a focus on gestation as productive of foetal bodies. This can silence alternative reproductive outcomes and obscure their value to re-thinking the relationships between gestating bodies and their boundaries. In this article, I have engaged with bioscientific and patient accounts of gestational trophoblastic disease and the hormone hCG to highlight pregnancy as a meaningful and productive event beyond foetal development.

In biomedicalised societies, hormones remain at the centre of explanations of who we are and how we reproduce (Roberts, 2007: 191). In this research, hCG, as articulated through a range of affective and technological practices, was key to establishing participants as pregnant. This hormone was often equated with a (potential) future baby by participants and also provided an explanation for embodied and emotional experiences such as ‘mood swings’ and nausea. As authors have shown in relation to sex hormones, the stories told about hCG both reflect and constitute dominant representations of the biological ‘facts’ of life (Oudshoorn, 1995; Roberts, 2007). Despite being just one of the many hormonal changes following conception, I argue that its positioning as ‘the pregnancy hormone’ shapes and is shaped by singular, teleological narratives of pregnancy that privilege foetal development. Its presence is straightforwardly equated with an anticipated future baby, socioculturally and within the clinical literature. However, in this research, I have shown the complexity of this association as played out within lived experience. Following Roberts (2007), patient accounts of hCG have shown that the hormone neither expresses nor produces bodies as pregnant, but is entangled with a range of personal, technological and clinical practices which shape its meaning for the users of home pregnancy tests, and its wider acceptance as the ‘pregnancy hormone’. Its flexibility as a material-semiotic object enabled some participants to counter dominant understandings of pregnancy through their accounts of the hormone in the context of gestational trophoblastic disease. Participants’ stories have therefore exemplified the ‘mutual coming into being of hormones and bodies’ (Sanabria, 2016: 187), with the same hormone-producing bodies which can be pregnant and/or enduring disease, and shifting its social meanings for participants like Lily from ‘good’ to ‘evil’.

Key to participants’ interpretations of hCG was its mode of materialisation. Home pregnancy tests are an ‘everyday’ technology and familiar within public consciousness (Olszynko-Gryn, 2017). Though offering autonomy and knowledge of a pregnancy privately (Leavitt, 2006), Layne (2009) has questioned the extent to which the pregnancy test can be considered a feminist technology. This is in part due to its privileging of technological ways of knowing over embodied experience, but also because the test is ‘universalist and reductionist’, able to diagnose only ‘a chemical pregnancy, not a physiological one’ (p. 66). However, participant accounts challenge this distinction between a chemical and a physiological pregnancy, with the chemical detection of hCG intimately tied with corporeal symptoms, each shaping the other. This was also evident within regimes of hCG testing for the purposes of monitoring disease, which could provoke ambiguous pregnant embodiment. Such experiences were heavily shaped by the modes of accessing this hormone. Surveillance was frequent and sometimes experienced as invasive to participants’ bodies and their environments. The quantification of hCG also impacted patients as they tracked their levels and waited anxiously for their results to fall. Previous work has considered the quantification of disease biomarkers, which can produce feelings of risk or vulnerability (Gillespie, 2012), but also reassurance by rendering disease knowable (Bell, 2013). This article has enhanced sociological understanding by attending to the mode of hCG’s materialisation, with its frequency and associated routines situating some within a ‘hamster wheel’ of monitoring, with implications for embodiment.

Embodied experience was at the heart of participant accounts. Bea’s queasiness and changes to her taste prompted her to take a pregnancy test, Juliet described lactation, with swollen bellies, nausea and tiredness also common. However, with teleological models of pregnancy dominating how they made sense of their experiences and shaping societal responses, some participants did not describe their embodied experiences as ‘pregnancy’, instead seeing themselves as undergoing a ‘false’ pregnancy or representing their bodies as having ‘tricked’ them. These perceptions stem from an understanding of pregnancy as defined by its future outcome, and not as a physiological, chemical and emotional process in its own right – an understanding stifled by foetal-centric approaches to pregnancy and its management in biomedicalised societies. This notion of pregnancy can also be seen in existing literature on pregnancy loss, which depicts miscarriage as the interruption of a ‘teleological passage towards childbirth’ and women experiencing loss as occupying a liminal state because they have not completed their pregnancy (Browne, 2022a: 106–107). Instead, this article has emphasised pregnancy as multiple and culminating in varying endpoints, not all of which entail foetal development. For participants undergoing monitoring for hCG, endpoints could even be surpassed and continually re-established. Some therefore challenged dominant narratives of gestation which did account for these more contingent and elusive experiences, by conceptually separating embodied pregnancy from foetal development. Clinical practitioners too emphasised the emotional and embodied in the management of gestational trophoblastic disease, recognising pregnancy as a significant event no matter the outcome, with this approach shaping clinical management.

My examples have demonstrated that ‘what makes a body pregnant’ can be located beyond foetal development or even beyond the gestating body, to the ‘hopes and dreams’ prompted by a welcomed positive pregnancy test, the relationships that share in their formation, and clinical practices that centre the patient. This inevitably produces multiple pregnancies with multiple outcomes, destabilising singular teleological models which continue to dominate regulatory practice and care.

## Conclusion

This article has contributed to feminist science studies perspectives which aim to de-privilege foetal bodies in understandings of pregnancy, extending this work by engaging with the rare case of gestational trophoblastic disease. Through personal accounts, I have shown that pregnancy must be appreciated as entailing multiple forms and as established through a range of chemical, technological and affective practices. As the quotes that opened this article show, pregnant bodies are not constituted by foetal development alone, but by developing placental tissues and hormones, technologies and more widely within subjectivities and relationships. However, despite diverse products and personal responses to pregnancy, one version, which privileges potential foetal futures, continues to dominate within reproductive healthcare and in laws governing access to such care. Feminist scholarship which destabilises dominant versions of pregnancy is vital in the face of increasing international restrictions on reproductive rights, and to ensure careful attention to lived experiences of this event in all its forms.

## Notes

1. The data presented here formed part of a research project led by the author, with the author undertaking all interviews, online data collection and analysis. Interviews with women affected by molar pregnancy were semi-structured and transcripts were analysed alongside blog posts by women affected, with codes developed and applied across the dataset as a whole in NVivo. Findings centring on clinical practice are being developed for publication elsewhere, but some data from interviews with practitioners are presented below. Fieldwork took place between January 2021 and October 2023, with ethical approval obtained from the University of Sheffield (037518), and an NHS Research Ethics Committee (21/WM/0018).
